# Influência do Exercício Físico sobre a Mecânica de Contração do Ventrículo Esquerdo após Infarto do Miocárdio

**DOI:** 10.36660/abc.20220185

**Published:** 2023-03-24

**Authors:** Márcio Silva Miguel Lima, Talia Falcão Dalçóquio, Maria Cristina Donadio Abduch, Jeane Mike Tsutsui, Wilson Mathias, José Carlos Nicolau

**Affiliations:** 1 Hospital das Clínicas Faculdade de Medicina Universidade de São Paulo São Paulo SP Brasil Instituto do Coração (InCor), Hospital das Clínicas HCFMUSP, Faculdade de Medicina, Universidade de São Paulo, São Paulo, SP – Brasil

**Keywords:** Infarto do Miocárdio, Exercício Físico, Disfunção Ventricular Esquerda

## Abstract

**Fundamento:**

O exercício exerce um papel positivo na evolução da doença cardíaca isquêmica, melhorando a capacidade funcional e prevenindo o remodelamento ventricular.

**Objetivo:**

Investigar o impacto do exercício sobre a mecânica de contração do ventrículo esquerdo (VE) após um infarto agudo do miocárdio (IAM) não complicado.

**Métodos:**

Um total de 53 pacientes foram incluídos e alocados aleatoriamente em um programa de treinamento supervisionado (grupo TREINO, n=27) ou em um grupo CONTROLE (n=26) que recebeu recomendações usuais sobre a prática de exercício físico após um IAM. Todos os pacientes realizaram um teste cardiopulmonar e um ecocardiograma com *speckle tracking* para medir vários parâmetros da mecânica de contração do VE em um mês e cinco meses após o IAM. Um valor de p <0,05 foi considerado para significância estatística nas comparações das variáveis.

**Resultados:**

Não foram encontradas diferenças nas análises dos parâmetros de *strain* circunferencial, radial ou longitudinal do VE entre os grupos após o período de treinamento. Após o programa, a análise da mecânica de torção revelou uma redução na rotação basal do VE no grupo TREINO em comparação ao grupo CONTROLE (5,9±2,3 vs. 7,5±2.9^o^; p=0,03), bem como na velocidade rotacional basal (53,6±18,4 vs. 68,8± 22,1 º/s; p=0,01), velocidade de twist (127,4±32,2 vs. 149,9±35,9 º/s; p=0,02) e na torção (2,4±0,4 vs. 2,8±0, º/cm; p=0,02).

**Conclusões:**

A atividade física não causou melhora significativa nos parâmetros de deformação longitudinal, radial ou circunferencial do VE. No entanto, o exercício teve um impacto significativo sobre a mecânica de torção do VE, que consistiu em uma redução na rotação basal, na velocidade de twist, na torção, e na velocidade de torção, que pode ser interpretada como uma “reserva” de torção ventricular nessa população.

**Figura Central:**
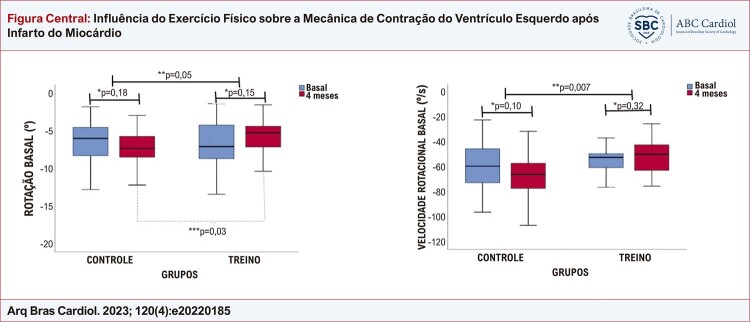
: Influência do Exercício Físico sobre a Mecânica de Contração do Ventrículo Esquerdo após Infarto do Miocárdio

Rotação basal do ventrículo esquerdo e velocidade rotacional; * teste t pareado; **teste t não pareado para comparação das variações entre os grupos após o seguimento; *** teste t não pareado. Figura original, criada pelos autores.

## Introdução

Os benefícios do exercício após um infarto agudo do miocárdio (IAM) têm sido investigados por muitos anos, e a maioria das publicações demonstraram um melhor prognóstico com essa prática.^1 - 8^ A atividade física após um IAM tem sido relacionada à prevenção do remodelamento cardíaco e da progressão da insuficiência cardíaca, e está associada com aumento da capacidade funcional.^4 , 9 - 13^ Esses fatores estão associados a um menor risco de IAM e redução na mortalidade e na internação por todas as causas.^14 , 15^

Tais benefícios têm sido demonstrado em casos de IAM com maior envolvimento contrátil do ventrículo esquerdo (VE), marcado por disfunção sistólica moderada e importante [fração de ejeção do ventrículo esquerdo (FEVE) ≤ 40% e ≤ 30%, respectivamente].^16 - 23^ Contudo, com a maior disseminação de informações quanto ao reconhecimento dos sintomas “de alerta”, maior rapidez no atendimento, evolução das terapias empregadas, tem-se observado um menor comprometimento após o IAM.^9^ Assim, a cardiomiopatia isquêmica com função sistólica do VE preservada ou disfunção leve (FEVE 41-51% em homens e 41-53% em mulheres) tem se tornado mais comum na população geral.^9^ Nesse contexto, pouco se conhece sobre os benefícios do exercício sobre o remodelamento do VE e os efeitos sobre os mecanismos de contração do VE nessa população.

A ecocardiografia com *speckle tracking* pode realizar um estudo abrangente da mecânica da contração do VE, caracterizada por um encurtamento longitudinal da base para o ápice, associado a rotações segmentares e torção ventricular.^24^ Essa análise fornece dados importantes que podem ser acrescidas à determinação da FEVE.^24 - 30^

O presente estudo tem como objetivo testar a hipótese de que a reabilitação cardíaca, promovida por um programa supervisionado de exercícios, teria um impacto sobre a mecânica da contração do VE em pacientes com IAM não complicado.

## Métodos

### Delineamento e população do estudo

Este foi um estudo prospectivo, longitudinal, randomizado e controlado. Pacientes com um IAM não complicado, admitidos na Unidade de Doença Coronariana Aguda do Instituto do Coração da Faculdade de Medicina da Universidade de São Paulo (InCor/HCFMUSP), que concordaram em participar do programa, foram separados aleatoriamente em dois grupos (TREINO e CONTROLE), na proporção de 1:1 de acordo com o seguinte protocolo: Tempo 0: durante internação, todos os pacientes, após devidas explicações, assinaram um termo de consentimento para inclusão no estudo; tempo 1: todos os participantes retornaram um mês após o IAM e foram submetidos a um ecocardiograma e a um teste cardiopulmonar (TCP). Em seguida, os participantes alocados no grupo TREINO foram incluídos em um programa de treino físico supervisionado, duas vezes por semana, por quatro meses, no Laboratório de Reabilitação, e os indivíduos alocados no grupo CONTROLE receberam recomendações usuais para a prática da atividade física na residência. Em resumo, os pacientes foram orientados à prática de exercícios aeróbicos de intensidade leve a moderada, por no mínimo 30 minutos, pelo menos três vezes por semana nos primeiros dois meses. No terceiro mês, os participantes foram orientados a aumentar a intensidade do exercício para moderada, três a cinco vezes por semana, 30 a 60 minutos. Não foi realizado monitoramento específico. O programa de treinamento supervisionado consistiu em: cinco minutos de exercício de alongamento, 40 minutos em uma bicicleta ergométrica, dez minutos de exercícios de fortalecimento, e cinco minutos de resfriamento com exercícios de alongamento. A intensidade do exercício foi estabelecida por níveis de frequência cardíaca correspondentes ao limitar anaeróbico e ao ponto de compensação respiratória. Tempo 2: ao final do quarto mês (quinto mês após o evento de IAM), todos os pacientes repetiram o ecocardiograma e o TCP ( material suplementar I ).

Os critérios de inclusão foram: idade acima de 18 anos, internação por IAM espontâneo com ou sem elevação do segmento ST, estabelecido de acordo com a terceira definição universal de IAM;^31^ pacientes estáveis clinicamente e hemodinamicamente; FEVE > 0,40 e classes Killip I ou II. Os critérios de exclusão foram: qualquer condição que contraindicava atividade física, praticantes de atividade física regular antes do evento (confirmado por entrevista para a inclusão); FEVE ≤ 40%; classe Killip III ou IV; ritmo cardíaco irregular (tais como fibrilação atrial, contrações atriais ou ventriculares prematuras frequentes); janela ecocardiográfica limitada para análise.

O presente estudo foi conduzido de acordo com a Declaração de Helsinki e aprovado pelo comitê de ética e pelo comitê científico da instituição. Todos os pacientes assinaram o termo de consentimento antes de serem incluídos no estudo.

### Ecocardiograma convencional

O ecocardiograma foi realizado por um único operador experiente, cego quanto ao grupo de alocação. Um aparelho de ecocardiografia comercialmente disponível (Vivid E9; GE Medical Systems, Milwaukee, WI, EUA) foi usado, equipado com transdutores lineares de banda larga, com uma frequência de 5-2 MHz. As medidas, análise dos fluxos valvares, e a avaliação da função diastólica do VE foram realizados seguindo-se diretrizes atuais.^32 - 35^

### Aquisição e análise das imagens por *speckle tracking*

Para a aquisição das imagens e análise subsequente pela técnica *speckle tracking* , o equipamento foi ajustado para aquisição de três ciclos cardíacos, 40-80 quadros por segundo. As imagens foram obtidas no corte paraesternal eixo curto do VE e seus três níveis principais: basal (valva mitral), medial (músculos papilares), e apical. A janela apical do VE foi composta pelo corte longitudinal, corte de duas câmaras e pelo corte de quatro câmaras.

**Figura 1:**
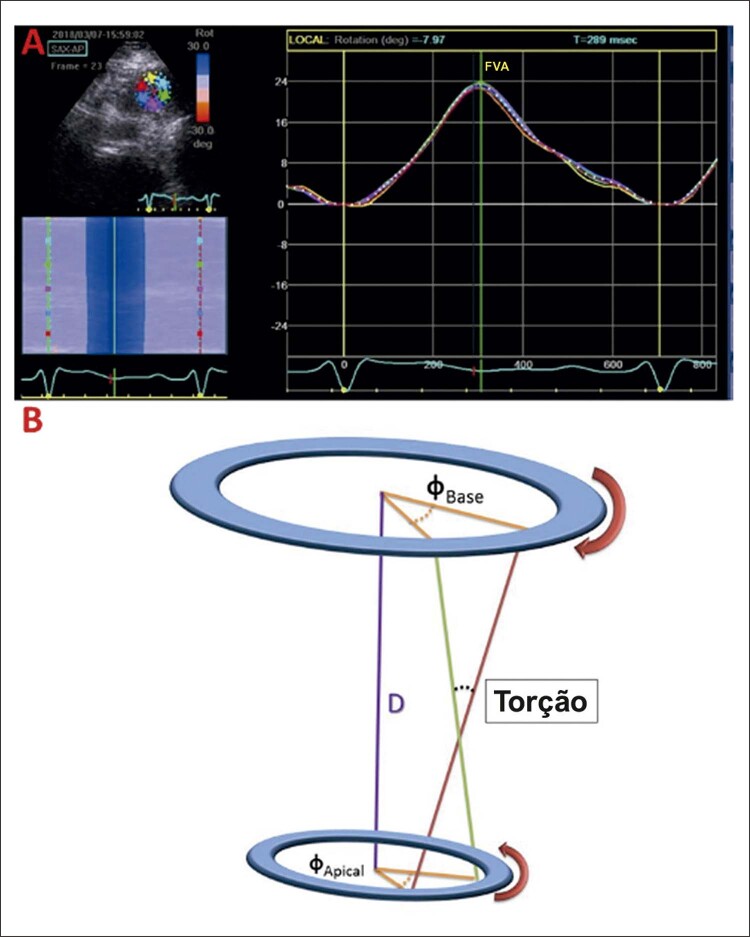
– A) exemplo de medida de rotação apical. B) representação gráfica da torção do ventrículo esquerdo; FVA: fechamento da valva aórtica.

Análises *off-line* foram realizadas usando o programa EchoPAC, versão v20.1 (GE Medical Systems, Milwaukee, WI, EUA). Os parâmetros avaliados foram: *strain* (ε,%) e *strain rate* (ε’,s^-1^); rotação basal e rotação apical máximas (VErot,^o^) e os picos de velocidades rotacionais (VErot-v,^o^/s), twist (VEtw,^o^) e torção do VE (VEtor,^o^/cm), e suas velocidades (VEtw-v,^o^/s; VEtor-v,^o^/s.cm). O twist ventricular esquerdo foi calculado como a diferença absoluta dos picos de rotação basal e apical (VEtw = apical VErot - basal VErot) e torção normalizada para o comprimento longitudinal do VE.^24^ Por convenção, os valores de rotação basais foram negativos, e os de rotação apical, positivos ( Figura 1 ).^24^ Neste artigo, os valores de *strain* negativos foram apresentados em módulos (positivos) para um melhor entendimento.

### Teste cardiopulmonar

O TCP foi realizado em um cicloergômetro eletromagnético (Medfit 400L, Medical Fitness Equipment, Maarn, Holanda), seguindo um protocolo de rampa, com uma velocidade de 60 rotações por minuto (rpm) e aumentos de carga de 10w a 20w por minuto, até atingir a exaustão física. Os participantes foram conectados a um ventilador (SensorMedics Corp, CA, EUA, e a ventilação pulmonar, concentração de oxigênio (O_2_), e concentração de dióxido de carbono (CO_2_) foram medidas para o cálculo do consumo de oxigênio (VO_2_) e produção de CO_2_. Quando o paciente atingia a exaustão, o limiar anaeróbico e o ponto de compensação respiratória (isto é, pontos quando o lactato sanguíneo começa a aumentar rapidamente devido à elevada intensidade no exercício e anaerobiose muscular) foram determinadas, ambos usados para prescrever a intensidade do treino.^36^

### Análise estatística

As variáveis contínuas foram apresentadas em média ± desvio padrão (DP) ou mediana e intervalos interquartis (IIQs), de acordo com a normalidade da distribuição avaliada pelo teste de Shapiro-Wilk. As variáveis categóricas foram apresentadas em números e porcentagens. Para o cálculo do tamanho amostral, considerou-se o valor médio do *strain* longitudinal global de 15,9% (±2,3).^37^ Para um ganho esperado de 10% no *strain* longitudinal no grupo TREINO, considerando o tamanho do efeito desta intervenção (d = 0,65), um poder (1 - β) de 80% para demonstrar diferenças, e um índice alfa de 0,05, foi necessário incluir 36 pacientes em cada grupo (tamanho amostral de 72 participantes). A randomização da amostra foi feita por meio do website “randomization.com”. Comparações das variáveis contínuas entre os grupos foram realizadas usando o teste t de *Student* (em caso de distribuição normal) ou o teste de Mann-Whitney (em caso de distribuição não normal) para amostras independentes. Para as variáveis categóricas, o teste do qui-quadrado (χ^2^) ou o teste exato de Fisher foi usado, conforme apropriado. A comparação intragrupo das médias foi realizada usando o teste t pareado. Por fim, a comparação do “delta” (Δ) dos valores obtidos do acompanhamento de cada grupo foi realizada pelo teste t não pareado.

A correlação linear de Pearson foi realizada para avaliar correlações entre variáveis do TCP e do ecocardiograma. Todos os testes foram bicaudais, e um valor de p<0,05 foi considerado estatisticamente significativo. A análise estatística foi realizada pelo programa SPSS v.25 para Macintosh (SPSS Inc, Chicago, IL). Análises entre observadores e intra-observador estão disponíveis no material suplementar II .

## Resultados

### Características clínicas

**Figura 2:**
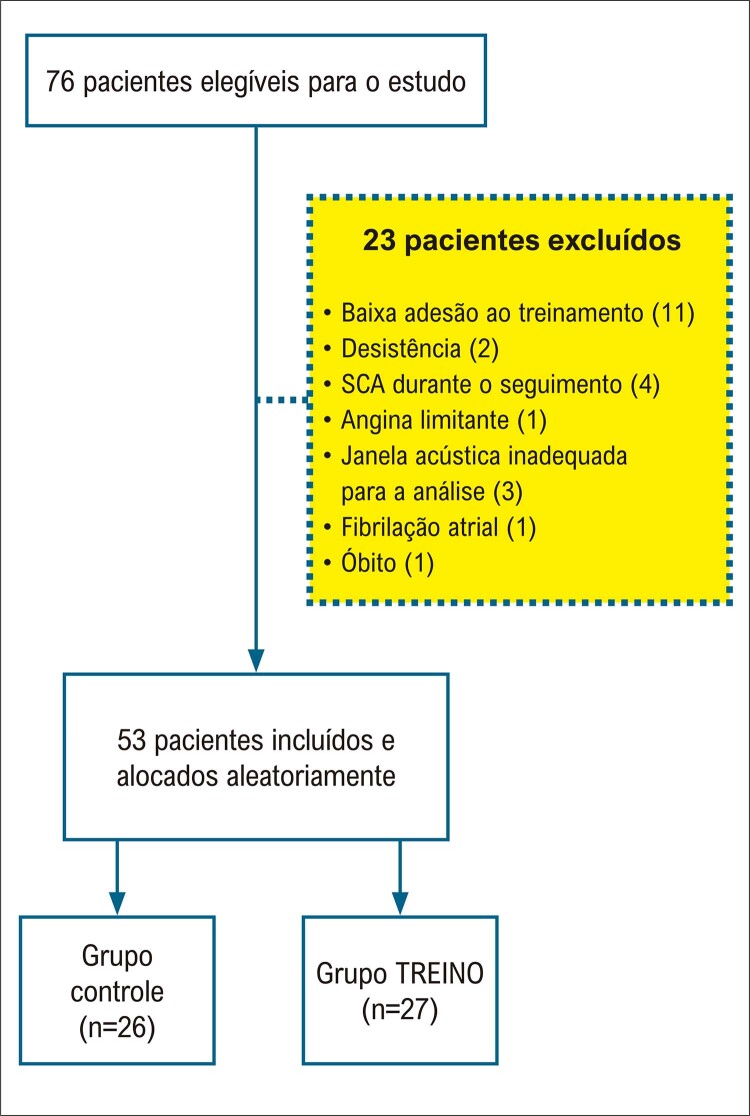
– Fluxograma de inclusão dos pacientes; SCA: síndrome coronariana aguda.

De 2016 a 2019, 76 pacientes foram recrutados. Desses, 23 foram excluídos por várias razões, sendo a baixa adesão ao exercício a principal ( Figura 2 ). A população final do estudo foi composta por 53 participantes. Após a randomização, 27 indivíduos foram incluídos no grupo TREINO e 26 no grupo CONTROLE.

As características da população estão apresentadas na Tabela 1 . Como pode ser observado, não houve diferenças estatisticamente significativas quanto à idade, sexo, variáveis relacionadas à doença arterial coronariana (DAC), tratamento hospitalar, risco e gravidade do evento isquêmico.

**Tabela 1:** – Características basais da população

Variáveis	CONTROLE (n = 26)	TREINO (n = 27)	p
**Demográficas**			
Idade, a	59±11	60±9	0,73
Sexo M / F, n (%)	19 (73%) / 7 (27%)	20 (74%) / 7 (26%)	0,93
IMC, Kg/m^2^	27,4±3,9	27,2±3,9	0,84
Hipertensão, n (%)	17 (65%)	13 (48%)	0,21
Diabetes Mellitus, n (%)	10 (38%)	6 (22%)	0,84
Tabagismo, n (%)	5 (19%)	10 (37%)	0,15
Sedentarismo, n (%)	26 (100%)	27 (100%)	1,00
Dislipidemia	8 (31%)	9 (33%)	0,84
IAM prévio, n (%)	4 (15%)	3 (11%)	0,70
**Tratamento**			
Aspirina, n (%)	26 (100%)	27 (100%)	1,00
Clopidogrel, n (%)	24 (92%)	27 (100%)	0,24
Ticagrelor, n (%)	2 (8%)	0 (0%)	0,24
β-bloqueador, n (%)	17 (65%)	23 (85%)	0,09
IECA/BRA	16 (61%)	21 (78%)	0,20
**Evento**			
IAMCSST , n (%)	14 (54%)	16 (59%)	0,51
IAMSSST, n (%)	12 (46%)	11 (41%)	0,69
Troponina, ng/mL (mediana, IIQ)	22 (3-50)	32 (11-50)	0,35
**Escores de risco**			
Escore IAMCSST TIMI	3,3±1,8	2,7±1,8	0,38
Escore IAMSSST TIMI	3,7±1,3	3,2±1,1	0,31
GRACE (mediana, IQR)	129 (120-159)	139 (121-151)	0,74
**Tratamento**			
Fibrinólise, n (%)	4 (15%)	6 (22%)	0,73
ATP primária, n (%)	8 (31%)	8 (30%)	0,93
Estratégia fármaco-invasiva, n (%)	3 (12%)	2 (7%)	0,66
SM, n (%)	19 (73%)	23 (85%)	0,23
SF, n (%)	5 (19%)	1 (4%)	0,10

*Dados expressos em média ± desvio padrão, mediana (IIQ, Intervalo Interquartil; IC 95%) ou número e porcentagens (%). Dados categóricos apresentados em números e porcentagens; IMC: índice de massa corporal; IECA: inibidor de enzima conversora de angiotensina; BRA: bloqueador de receptor de angiotensina; IAMCSST: infarto do miocárdio com supradesnivelamento do segmento ST; TIMI: trombólise no infarto do miocárdio; IAMSSST: infarto do miocárdio sem supradesnivelamento do segmento ST; GRACE: Global Registry of Acute Coronary Events Score; ATP: angioplastia transluminal percutânea; SM: stent metálico; SF: stent farmacológico. P não significativo para todas as comparações (teste t de Student, teste do qui-quadrado ou teste exato de Fisher).*

A adesão média dos pacientes no grupo TREINO ao programa de exercício foi de 28,0 ± 6,8 sessões (15 - 39 sessões), alcançando uma adesão global de 88%.

### Teste cardiopulmonar

A Tabela 2 apresenta os principais dados do TCP. Após o programa de exercício, o grupo TREINO mostrou um aumento significativo na duração do exercício, carga máxima atingida e pico de VO_2_. Contudo, ao final do período de acompanhamento de quatro meses, não foram observadas diferenças nas variações [delta (Δ)] dos parâmetros medidos entre os grupos ( material suplementar III ).

**Tabela 2:** – Variáveis ecocardiográficas e do teste cardiopulmonar

Variáveis	CONTROLE			TREINO		
	
	Basal	4M	p*	Basal	4M	p*
**Ecocardiograma**						
AE, mm	37,3±3,7	36,6±3,4	0,02	38,7±3,4	38,8±3,3	0,63
DDFVE, mm	50,2±4,3	50,0±4,3	0,21	50,9±3,1	51,3±3,5	0,38
DSFVE, mm	33,1±5,0	31,9±3,9	0,05	33,6±3,0	34,6±4,6	0,11
VDFVE, ml	99,9±23,2	99,5±23,5	0,66	94,3±15,2	92,2±16,4	0,40
VSFVE, ml	39,6±11,1	42,0±10,2	0,11	42,3±11,5	42,6±9,1	0,85
IMVE, g/m^2^	90,7±18,6	89,7±18,0	0,28	92,7±16,7	92,2±16,4	0,88
FEVE, %	61,3±5,7	60,0±5,1	0,19	60,0±5,9	59,6±5,6	0,70
IEMP	1,19±0,19	1,16±0,19	0,19	1,18±0,16	1,17±0,18	0,39
Vel E, cm/s	81±24	81±22	0,89	74±0,18	74±18	0,95
2Vel e’, cm/s	7,1±1,6	7,2±1,8	0,80	6,9±1,7	6,9±1,8	0,89
2Razão E/e’	12,1±5,3	11,8±4,6	0,65	11,5±3,9	11,6±4,9	0,92
Razão E/A	1,21±0,52	1,13±0,39	0,34	1,09±0,41	1,20±0,46	0,16
**TCP**						
Duração do exercício, s	491±93‡	593±93	0,006	524±95‡	636±131	0,001
FC Basal, bpm	73±14	70±11	0,58	70±10	64±10	0,002
FC Max, bpm	133±22	134±15	0,84	127±19	128±19	0,78
PAS basal, mmHg	124±11	119±14	0,35	120±18	117±14	0,51
PAS pico, mmHg	169±18†	176±16	0,41	184±27†	178±18	0,41
Carga máxima de exercício, W	109±43	119±48	0,11	126±49	151±65	0,007
VO_2_ pico, ml/kg/min	20,6±4,6	21,7±4,9	0,12	21,7±5,2	23,4±5,9	0,04
LA, mL/kg/min	12,3±2,3	13,7±2,4	0,08	12,3±3,2	14,3±3,4	0,11
VM, L/min	59,5±21,3	59,4±19,5	0,97	62,7±18,7	69,2±20,4	0,93

Dados expressos em média ± DP; AE: átrio esquerdo; DDFVE: diâmetro diastólico final do ventrículo esquerdo; DSFVE: diâmetro sistólico final do ventrículo esquerdo; VDFVE: volume diastólico final do ventrículo esquerdo; VSFVE: volume sistólico final do ventrículo esquerdo; FEVE: fração de ejeção do ventrículo esquerdo; IEMP: índice do escore de motilidade de parede; IMVE: índice da massa ventricular esquerda; Vel E: velocidade da onda E transmitral; Vel e’: velocidade da onda e’ septal; FC: frequência cardíaca; s: segundos; PAS: pressão arterial sistólica; VO_*2*_ : consumo de oxigênio; LA: limiar anaeróbico; VM: volume minuto; * teste t pareado. ‡p=0,02, teste t não pareado, comparação com CONTROLE basal. †p=0,04, teste t não pareado, comparação com CONTROLE basal.

### Ecocardiograma convencional

Os dados ecocardiográficos estão apresentados na Tabela 2 . Uma pequena diferença nas dimensões do átrio esquerdo foi observada no grupo CONTROLE no quarto mês. Não foram encontradas outras diferenças significativas, incluindo comparações nas variações nesse parâmetro.

### Análise da mecânica na contração do VE

#### Corte apical

#### Strain e strain radial (SR)

Não foram observadas diferenças significativas no *strain* ou SR (strain radial) em resposta ao exercício entre os grupos ao final do seguimento ( Tabela 3 e material suplementar IV ).

**Tabela 3:** – Resultado da análise do strain e do strain rate do ventrículo esquerdo; análise da janela apical (cortes longitudinal, de quatro e de duas câmaras)

Variáveis	CONTROLE			TREINO		
	
	Basal	4 meses	p*	Basal	4 meses	p*
**APLAX**						
SL, %	17,5 ± 3,3	18,6 ± 2,5	0,09	17,6 ± 2,0	17,8 ± 2,8	0,54
2SRL, 1/s	0,98 ± 0,16	1,04 ± 0,20	0,08	0,97 ± 0,15	0,95 ± 0,20	0,65
**A4C**						
SL, %	18,5 ± 3,3	18,6 ± 2,8	0,91	17,9 ± 2,7	17,8 ± 3,3	0,89
SRL, 1/s	1,01 ± 0,23	1,00 ± 0,20	0,98	0,93 ± 0,16	0,90 ± 0,21	0,44
**A2C**						
SL, %	18,7 ± 2,9	18,4 ± 2,7	0,36	19,4 ± 2,8	18,4 ± 3,4	0,06
SRL, 1/s	1,04 ± 0,21	1,01 ± 0,15	0,24	1,03 ± 0,17	0,92 ± 0,18	0,01
**GLOBAL**						
SL, %	18,3 ± 2,7	18,5 ± 2,5	0,55	18,3 ± 2,2	18,0 ± 2,9	0,48
SRL, 1/s	1,00 ± 0,18	1,02 ± 0,17	0,67	0,98 ± 0,14	0,92 ± 0,18	0,14

Dados expressos em média ± desvio padrão; APLAX: janela apical, corte longitudinal; A4C: janela apical, corte de quatro câmaras; A2C: janela apical, corte de duas câmaras; SL: strain longitudinal; SRL: strain rate longitudinal; P não significativo para todas as comparações (teste t de Student); *teste t pareado

#### Eixo transversal

#### *Strain* , SR e rotação circunferencial e radial

Os dados obtidos da análise do eixo transversal do VE estão apresentados na Tabela 4 . Não foram observadas diferenças significativas na variação dos valores de *strain* ou SR e circunferencial entre os grupos após o seguimento. Em relação à mecânica rotacional, no final do período de treinamento, o grupo TREINO apresentou valores mais baixos de rotação basal em comparação ao grupo CONTROLE (TREINO 4M, -5,9±2,3 vs. CONTROLE 4M, -7,5 ± 2,9^o^; p=0,03), com o delta desse parâmetro apresentando uma significância *borderline* (p=0,05) entre os grupos. Ainda, valores mais baixos de velocidade de rotação basal foram observados no grupo TREINO (-53,6±18,4 vs.-68,8 ± 22,1º/s; p=0,01), conforme apresentado na ilustração central e material suplementar V .

**Tabela 4:** – Análise da mecânica da contração ventricular obtida no eixo curto transversal do ventrículo esquerdo (basal, medial e apical) e twist e torção do ventrículo esquerdo

	CONTROLE			TREINO		
	
	Basal	4 meses	p*	Basal	4 meses	p*
**PSAX – Basal**						
SC, %	17,0 ± 3,9	18,0 ± 3,1	0,11	16,6 ± 2,6	16,1 ± 3,4	0,38
SR, %	39,7 ± 21,0	34,3 ± 17,9	0,13	34,8 ± 13,9	31,8 ± 15,7	0,41
SRC, 1/s	1,54 ± 0,40	1,62 ± 0,36	0,42	1,52 ± 0,36	1,47 ± 0,31	0,58
SRR, 1/s	2,32 ± 0,92	2,41 ± 0,83€	0,68	2,20 ± 0,71	1,87 ± 0,71€	0,03
Rot, ^o^	-6,7 ± 3,2	-7,5 ± 2,9†	0,18	-6,7 ± 2,9	-5,9 ± 2,3†	0,15
Vel rot, ^o^/s	-66,6 ± 17,9	-68,8 ± 22,1‡	0,10	-57,8 ± 13,8	-53,6 ± 18,4‡	0,32
**PSAX – Médio**						
SC, %	15,8 ± 4,1	16,3 ± 3,6	0,55	15,9 ± 3,2	16,1 ± 3,8	0,71
SR, %	34,4 ± 19,3	39,4 ± 18,0	0,24	34,4 ± 11,9	35,6 ± 18,3	0,75
SRC, 1/s	1,44 ± 0,25	1,43 ± 0,26	0,75	1,43 ± 0,25	1,41 ± 0,31	0,48
SRR, 1/s	2,10 ± 0,84	2,31 ± 1,11	0,23	1,95 ± 0,58	2,16 ± 0,89	0,87
**PSAX – Apical**						
SC, %	19,3 ± 4,8	18,0 ± 3,9	0,16	20,5 ± 5,9	19,7 ± 5,8	0,62
SR, %	23,9 ± 9,0	19,1 ± 8,2	0,19	22,5 ± 7,7	25,0 ± 11,9	0,43
SRC, 1/s	1,53 ± 0,40	1,48 ± 0,42	0,67	1,56 ± 0,38	1,36 ± 0,39	0,09
SRR, 1/s	1,89 ± 0,97	1,80 ± 0,91	0,77	1,73 ± 1,18	1,90 ± 1,32	0,52
Rot, ^o^	15,5 ± 6,5	15,5 ± 5,0	0,95	14,9 ± 5,6	14,6 ± 4,1	0,74
2Vel rot, ^o^/s	84,9 ± 26,8	81,1 ± 23,2	0,43	78,0 ± 21,7	73,8 ± 18,7	0,38
**GLOBAL**						
SC, %	17,4 ± 3,5	17,4 ± 2,6	0,95	17,7 ± 3,1	17,3 ± 3,6	0,62
SR, %	30,8 ± 12,7	30,7 ± 8,5	0,94	29,5 ± 8,8	30,1 ± 12,7	0,78
SRC, 1/s	1,51 ± 0,27	1,51 ± 0,21	0,97	1,51 ± 0,26	1,41 ± 0,27	0,21
SRR, 1/s	2,10 ± 0,58	2,17 ± 0,52	0,57	1,96 ± 0,47	1,98 ± 0,74	0,92
**TORSION**						
*Twist* , ^o^	22,2 ± 7,1	23,1 ± 6,0	0,46	21,6 ± 6,2	20,5 ± 3,9	0,41
Vel Tw, ^o^/s	145,5 ± 32,3	149,9 ± 35,9¥	0,53	135,8 ± 28,3	127,4 ± 32,2¥	0,28
Torção, ^o^/cm	2,6 ± 0,8	2,8 ± 0,8£	0,18	2,6 ± 0,7	2,4 ± 0,4£	0,27
Vel T, ^o^/s.cm	17,4 ± 3,9	18,5 ± 5,2	0,22	16,3 ± 3,6	15,0 ± 3,6	0,17

Dados expressos em média ± desvio padrão. PSAX-basal: corte paraesternal eixo curto basal; PSAX-mid: corte paraesternal eixo curto medial; PSAX-apical: corte paraesternal eixo curto apical; SC: strain circunferencial; SR: strain radial; SRC: strain rate circunferencial; SRR: strain rate radial; Rot: rotação; Vel Rot: velocidade rotacional; Vel Tw: velocidade de twist; vel T: velocidade de torção. *Teste t pareado. €p=0,01, teste t não pareado, em comparação com CONTROLE 4 MESES. †p=0,03, teste t não pareado, em comparação com CONTROLE 4 MESES. ‡p=0,01, teste t não pareado, em comparação com CONTROLE 4 MESES. ¥p=0,02, teste t não pareado, em comparação com CONTROLE 4 MESES. £p=0,02, teste t não pareado, em comparação com CONTROLE 4 MESES.

## Twist e torção do VE

Resultados das análises do twist a da torção do VE estão apresentados na Tabela 4 . Ao final do quarto mês, o grupo TREINO apresentou valores significativamente mais baixos da velocidade de twist (127,4±32,2 vs.149,9±35,9 º/s; p=0,02) e de torção (2,4±0,4 vs. 2,8±0,8 º/cm; p=0,02). Contudo, não foram observadas diferenças estatisticamente significativas em nenhum dos deltas nos parâmetros da mecânica de torção entre os grupos ( material suplementar VI ).

## Análise de correlação

A análise de correlação linear de Pearson foi realizada para avaliar a correlação entre os deltas de VO_2_ (quatro meses em relação ao basal) obtida do TCP e os deltas de vários parâmetros ecocardiográficos. Não foram observadas correlação estatisticamente significativas ( material suplementar VII ).

## Discussão

O presente estudo investigou a hipótese de que a reabilitação cardiovascular, por meio de um programa de exercício supervisionado, teria um impacto sobre a mecânica de contração do VE em uma população após um IAM não complicado. Há poucos estudos investigando o real benefício do exercício sobre o remodelamento do VE, com análise da mecânica de contração do VE nessa população. Até o momento, em nosso conhecimento, não existe estudo similar investigando essa hipótese de maneira tão detalhada, considerando o grande número de parâmetros de contração do VE, incluindo os de mecânica da função sistólica, analisados em nosso estudo. Neste estudo, utilizamos a técnica de *speckle tracking* na investigação da função sistólica do VE. Esse método tem se demonstrado superior à FEVE, por ser menos susceptível às condições hemodinâmicas, mais reproduzíveis,^38^ e também um melhor definidor prognóstico para eventos cardiovasculares em um amplo espectro de doenças cardíacas.^39 - 41^ Tal fato é de particular importância após um evento de isquemia miocárdica em que uma análise precisa da função sistólica do VE é essencial.

Quanto às deformações longitudinal, circunferencial e radial do miocárdio, em geral, o grupo TREINO não apresentou um desempenho superior de contração em comparação ao grupo CONTROLE até quatro meses após o IAM. Embora o grupo TREINO tenha mostrado um aumento significativo na duração do exercício, carga máxima alcançada e pico de VO_2_ após o período de treinamento, não foi observado aumento na mecânica longitudinal ou transversal do VE. Contudo, identificamos um resultado muito interessante em relação à mecânica de torção. Comparativamente ao grupo controle, o grupo TREINO mostrou valores significativamente mais baixos de rotação e de velocidade de rotação dos segmentos basais do VE, bem como valores mais baixos de torção, e de velocidade de twist e de torção após as 16 semanas de treinamento. McGregor et al.^42^ observaram resultados similares em seu elegante estudo exploratório.^42^ Esses autores também descreveram uma redução no twist e na velocidade de twist após 10 semanas de sessões de treinamento físico, duas vezes por semana, em uma população similar que sofreu um IAM e manteve a função ventricular esquerda preservada (FEVE > 50%). Em seu estudo, esse resultado final relacionado ao twist do VE foi associado a uma redução tanto na rotação basal como na rotação apical. Finalmente, similar ao nosso estudo, os autores não encontraram um impacto positivo significativo do exercício sobre o *strain* do VE (longitudinal, circunferencial ou radial).

Extrapolando para atletas de alto rendimento, apesar de controvérsias nos achados, os estudos apontam para um impacto real final e comum do exercício sobre a mecânica de torção do VE. Stöhr et al. descreveram uma redução da rotação apical e do twist do VE em indivíduos com alta capacidade aeróbica.^43^ O mesmo foi observado por Nottin et al.,^44^ estudando ciclistas de elite, e Zócalo et al.^45^ avaliando jogadores de futebol profissionais. Foi descrita uma redução nas velocidades de rotação apical e basal do VE, e na velocidade de torção. Weiner et al.^46^ também descreveram achados interessantes com atletas de remo de competição. Em um programa de atividade física de alto nível, os autores descreveram um “fenômeno fásico”, compreendendo uma fase aguda de aumento no twist do VE, seguido de uma redução subsequente crônica nesse parâmetro.^46^ Com base nesse fato, é válido supor que um maior aumento no twist do VE durante o exercício pode representar maior eficiência sistólica nesses indivíduos. Esse desfecho pode ser interpretado como uma “reserva” de torção do VE em atletas e em indivíduos que se exercitaram após IAM, representando uma mecânica fisiologicamente mais eficiente, o que levaria a um aumento na capacidade funcional, e possivelmente a uma melhor evolução clínica.

O delineamento mais aceito da arquitetura do músculo cardíaco é o proposto por Torrent-Guasp et al.,^47 , 48^ que descreveram o coração como uma banda muscular “dobrada” em dupla hélice. Em termos de gasto energético, tal arquitetura provê uma forma mais eficiente de contração, bem como uma distribuição mais homogênea do estresse da parede, com menos consumo de oxigênio pelo miocárdio, em comparação a uma simples deformação da cavidade do VE.^49^

O efeito do exercício sobre o *strain* do VE ainda não está claro. Em uma revisão sistemática e meta-análise, Murray et al.^50^ estudaram o efeito do exercício sobre o *strain* longitudinal global do VE em uma ampla gama de populações sadias, em risco de doença cardiovascular, e com doenças crônicas. Naqueles com doença cardíaca, observou-se um efeito moderado do exercício em comparação a controles não praticantes de atividade física, e não houve efeito significativo do exercício nos indivíduos sadios ou em risco de doença cardiovascular em comparação aos controles que não praticaram atividade física. Similar ao nosso estudo, McGregor et al.^42^ não encontraram um impacto positivo do exercício sobre o *strain* longitudinal. Assim, apesar da boa acurácia e dados mais robustos na literatura sobre o *strain* longitudinal, o twist do VE poderia ser um parâmetro mais sensível para avaliar a resposta sistólica global do VE ao exercício físico nessa população.

### Limitações

Primeiro, o tamanho relativamente pequeno da amostra, a curta duração do programa de exercício, o pequeno número de sessões de treinamento para os pacientes no grupo TREINO (duas vezes por semana), e a baixa adesão de alguns pacientes ao programa pode ter diminuído o poder do estudo em demonstrar possíveis diferenças entre grupos. Os indivíduos eram incentivados a participar das sessões e, aqueles que compareceram a menos de uma sessão por semana, ou pararam as sessões, foram excluídos. Um tamanho amostral de 72 participantes, como calculado, não foi alcançado, o que consiste em mais uma limitação. Ainda, além da prática de exercícios, não foi dada nenhuma outra orientação relacionada à saúde, como suporte dietético ou psicológico, o que poderia ter afetado o resultado final. Não foram coletados dados sobre síndromes coronárias crônicas ou doença arterial periférica, que poderiam ter tido um impacto sobre o exercício. Por fim, nem todos os dados derivados do TCP como dados sobre a taxa de troca respiratória, foram abordados neste estudo, o que poderia ter revelado diferenças entre os grupos.

Outro aspecto importante é a subjetividade do ecocardiograma, que pode levar ao viés de quantificação. Pequenas variações na aquisição no nível apical do VE, que não possui um marcador anatômico, podem resultar na distorção dos valores. Neste sentido, criamos outro critério para confirmar uma aquisição correta, que consistiu na visualização de pelo menos uma tendência a uma rotação anti-horário dos segmentos, o que era fisiologicamente esperado. Finalmente, é importante lembrar que o examinador era cego para a alocação dos pacientes nos grupos.

## Conclusão

Em nosso estudo, o exercício não causou melhora significativa nos parâmetros de deformação longitudinal, radial ou circunferencial. Contudo, o exercício foi associado a uma redução na rotação basal, na velocidade de twist, na torção e na velocidade de torção. Esse desfecho pode ser interpretado como uma “reserva” da torção do VE em indivíduos que se exercitaram após o IAM, sugerindo uma mecânica de torção fisiologicamente mais eficiente.

## * Material suplementar

Para informação adicional do Material Suplementar 1, por favor, clique aqui  .

Para informação adicional do Material Suplementar 2, por favor, clique aqui  .

Para informação adicional do Material Suplementar 3, por favor, clique aqui  .

Para informação adicional do Material Suplementar 4, por favor, clique aqui  .

Para informação adicional do Material Suplementar 5, por favor, clique aqui  .

Para informação adicional do Material Suplementar 6, por favor, clique aqui  .

Para informação adicional do Material Suplementar 7, por favor, clique aqui  .
