# Supplementary material for: Influência do Exercício Físico sobre a Mecânica de Contração do Ventrículo Esquerdo após Infarto do Miocárdio
Source: Arq Bras Cardiol. 2023 Mar 24;120(4):e20220185. [Article in Portuguese] doi: 10.36660/abc.20220185 (PMC10263431; doi:10.36660/abc.20220185)
Supplement: Translation

# Translation: Influence of Physical Training after a Myocardial Infarction on Left Ventricular Contraction Mechanics

**Authors:** Márcio Silva Miguel Lima, Talia Falcão Dalçóquio, Maria Cristina Donadio Abduch, Jeane Mike Tsutsui, Wilson Mathias, José Carlos Nicolau

**Affiliations:** 1 Brazil Instituto do Coração (InCor), Hospital das Clínicas HCFMUSP, Faculdade de Medicina, Universidade de São Paulo, São Paulo, SP – Brazil

**Keywords:** Myocardial Infarction, Exercise, Left Ventricular Dysfunction

## Abstract

**Background:**

Exercise plays a positive role in the course of the ischemic heart disease, enhancing functional capacity and preventing ventricular remodeling.

**Objective:**

To investigate the impact of exercise on left ventricular (LV) contraction mechanics after an uncomplicated acute myocardial infarction (AMI).

**Methods:**

A total of 53 patients was included, 27 of whom were randomized to a supervised training program (TRAINING group), and 26 to a CONTROL group, who received usual recommendations on physical exercise after AMI. All patients underwent cardiopulmonary stress testing and a speckle tracking echocardiography to measure several parameters of LV contraction mechanics at one month and five months after AMI. A p value < 0.05 was considered statistically significant for the comparisons of the variables.

**Results:**

No significant difference were found in the analysis of LV longitudinal, radial and circumferential strain parameters between groups after the training period. After the training program, analysis of torsional mechanics demonstrated a reduction in the LV basal rotation in the TRAINING group in comparison to the CONTROL group (5.9±2.3 vs. 7.5±2.9^o^; p=0.03), and in the basal rotational velocity (53.6±18.4 vs.68.8±22.1 º/s; p=0.01), twist velocity (127.4±32.2 vs. 149.9±35.9 º/s; p=0.02) and torsion (2.4±0.4 vs. 2.8±0.8 º/cm; p=0.02).

**Conclusions:**

Physical activity did not cause a significant improvement in LV longitudinal, radial and circumferential deformation parameters. However, the exercise had a significant impact on the LV torsional mechanics, consisting of a reduction in basal rotation, twist velocity, torsion and torsional velocity which can be interpreted as a ventricular “torsion reserve” in this population.

## Introduction

The benefits of exercise after acute myocardial infarction (AMI) have been investigated for several years and most publications have demonstrated a better prognosis related to this practice.^1 - 8^ Physical activity post-AMI has been related to prevention of cardiac remodeling and of heart failure progression, and associated with increased functional capacity.^4 , 9 - 13^ These are linked to lower risk of AMI and reduction in all-cause hospitalization and mortality.^14 , 15^

Benefits have been demonstrated in cases of AMI with greater impairment of LV contraction, causing moderate and severe systolic dysfunction (left ventricular ejection fraction [LVEF] ≤ 40% and ≤ 30%, respectively).^16 - 23^ However, with broader dissemination of information regarding the recognition of AMI “alarm symptoms”, greater promptness of care, evolution of the therapies employed, less impairment of cardiac contraction after AMI has become more frequent.^9^ So, ischemic cardiomyopathy with preserved left ventricular (LV) systolic function or mild dysfunction (LVEF 41-51% in men and 41-53% in women) has become more frequent in the general population.^9^ In this context, little is known about the overall benefit of exercise on LV remodeling and the effects on the mechanisms of left ventricular contraction in this population.

Speckle tracking echocardiography (STE) can perform a comprehensive study of LV contraction mechanics, which is characterized by longitudinal apex-to-base shortening in association to segmental rotations and ventricular torsion.^24^ This analysis adds important data to the simple determination of LVEF.^24 - 30^

The present study aims to test the hypothesis that cardiovascular rehabilitation, promoted by a supervised exercise program, would have a positive impact on the mechanics of LV contraction in patients with uncomplicated AMI.

## Methods

### Study design and population

This was a prospective, longitudinal, randomized and controlled study. Patients with uncomplicated AMI admitted to the Acute Coronary Disease Unit of the Heart Institute of the University of Sao Paulo Medical School (InCor/HCFMUSP), who agreed to participate in the study, were randomized to two groups (TRAINING and CONTROL), in the proportion 1:1 according to the following protocol: Time 0: still hospitalized, all patients, after due explanations, signed the informed consent for inclusion in the study. Time 1: all participants returned one month after AMI and were submitted to an echocardiogram and cardiopulmonary exercising testing (CPX). After this, participants randomized to the TRAINING group were included in a supervised physical training program, twice a week, for four months, at the Rehabilitation Lab, while individuals randomized to the CONTROL group received usual recommendations for physical activity at home. Briefly, patients were instructed to engage in aerobic exercises of low-to-moderate intensity, with a minimum duration of 30 minutes, at least three times a week in the first two months. In the third month, they were instructed to increase the intensity to a moderate level 3-5 times a week, 30 to 60 minutes. No specific monitoring was carried out. Supervised training program consisted of two 60-minute exercise sessions per week. Each exercise session consisted of 5 minutes of stretching exercises, 40 minutes of cycling on a stationary bicycle, 10 minutes of local strengthening exercises, and five minutes of cool down with stretching exercises. The exercise intensity was established by heart rate levels that corresponded to the anaerobic threshold and respiratory compensation point. Time 2: at the end of the fourth month (fifth month after the AMI event), all patients repeated the echocardiogram and CPX (supplementary data I).

Inclusion criteria were: age over 18; hospitalization for spontaneous AMI with or without ST-segment elevation, established according to the 3^rd^Universal Definition of AMI;^31^ clinically and hemodynamically stable patients; LVEF > 0.40 and Killip classes I or II. The exclusion criteria were: any condition that contraindicated physical activity; regular physical activity practitioners prior to the event (confirmed by interview for inclusion); LVEF ≤ 40%; Killip classes III or IV; irregular heart rhythm (such as atrial fibrillation, frequent premature atrial or ventricular contractions); limited echocardiographic window for analysis.

The present study was conducted in accordance with the Declaration of Helsinki and was approved by the institutional ethics and scientific committees. All the patients signed the informed consent form before inclusion in the study.

### Conventional echocardiogram

The echocardiogram was performed by a single experienced operator blind to the allocation group. Commercially available echocardiographic equipment (Vivid E9; GE Medical Systems, Milwaukee, WI, USA) was used, equipped with linear broadband transducers with a frequency of 5-2 MHz. Measurements, and analysis of valve flow and LV diastolic function were performed according to current guidelines.^32 - 35^

### STE image acquisition and analysis

For image acquisition for speckle tracking analysis, the machine was adjusted to acquire three cardiac cycles, with frame rate between 40-80 frames/s. Images were obtained in the parasternal short axis (PSAX) view in its three main levels: basal (mitral valve), mid (papillary muscles) and apical. LV apical window was composed by: longitudinal (APLAX), four (A4C) and two chambers (A2C) views.

Off-line analyses were performed using the EchoPAC software, version v20.1 (GE Medical Systems, Milwaukee, WI, USA). The parameters evaluated were: strain (ε,%) and strain rate (SR) (ε’,s^-1^); maximum basal and apical rotations (LVrot,^o^) and their peak absolute rotational velocities (LVrot-v,^o^/s), LV twist (LVtw,^o^) and torsion (LVtor,^o^/cm), and their velocities (LVtw-v,^o^/s; LVtor-v,^o^/s.cm). LV twist was calculated as the absolute difference between peak systolic apical and basal rotations (LVtw = apical LVrot - basal LVrot) and torsion as the twist normalized to LV longitudinal length.^24^ By convention, baseline rotation values were negative, and apical, positive ( Figure 1 ).^24^ In this paper, the negative strain values will be expressed as modulus (positive) for a better understanding.

**Figure 1:** – A) example of apical rotation measurement. B) graphical representation of left ventricular torsion. AVC: aortic valve closure.

### Cardiopulmonary exercise testing

The CPX was performed on an electromagnetic cycle ergometer (Medfit 400L, Medical Fitness Equipment, Maarn, Netherlands), following a ramp protocol, with a speed of 60 revolutions per minute (rpm) and workload increments from 10w to 20w per minute, until achieving physical exhaustion. Participants were connected to a ventilator (SensorMedics Corp, CA, USA), and pulmonary ventilation, oxygen (O_2_) and carbon dioxide (CO_2_) concentrations were measured with calculation of oxygen consumption (VO_2_) and CO_2_production. When the patient achieved exhaustion, the anaerobic threshold and respiratory compensation point (i.e. points when blood lactate starts to increase rapidly due to high exercise intensity and tissue anaerobiosis) were determined, both used to prescribe the intensity of physical training.^36^

### Statistical analysis

Continuous data are presented as mean ± standard deviation (SD) or median and interquartile ranges (IQR), according to normality of distribution assessed by the Shapiro-Wilk test. Categorical data were presented as numbers and percentages. To calculate the sample size, the mean value of global longitudinal strain of 15.9% (± 2.3) was considered.^37^ For an expected gain of 10% in longitudinal strain in the TRAINING group, considering the effect size of this intervention (d = 0.65), a power (1 - β) 0f 80% to detect differences and alpha index of 0.05, it was necessary to include 36 patients in each group (a sample size of 72 participants). Randomization of the sample was made through the website “randomization.com”. Comparisons of continuous variables between groups were performed using the Student’s t-test (in case of normal distribution) or Mann-Whitney test (in case of non-normal distribution) for independent samples. For categorical variables, the Chi-square test (χ^2^) or Fisher’s exact test was used, as appropriate. The within-group comparison of means was performed using the paired Student’s t test. Finally, comparison of the “delta” (Δ) of values obtained from the follow-up of each group was done by the unpaired Student’s t test.

### Pearson’s linear correlation was performed to assess correlations between CPX and echocardiographic variables.

All tests were 2-tailed and a p value <0.05 was considered statistically significant. Statistical analysis was done using the SPSS v.25.0 software for Macintosh (SPSS Inc, Chicago, IL). Inter and intra-observer analyses and Bland-Altman plots are disposed in the supplementary data II.

## Results

### Clinical characteristics

From 2016 to 2019, a total of 76 patients was enrolled. Of these, 23 were excluded for several reasons, with poor adherence to exercise as the main reason ( Figure 2 ). The final study population consisted of 53 participants. After randomization, 27 individuals were included in the TRAINING group and 26 in the CONTROL group.

**Figure 2:** – Patient inclusion flowchart. ACS: acute coronary syndrome.

The clinical characteristics of the population are shown in Table 1 . As can be seen, there was no significant differences between the groups regarding age, sex, variables related to coronary artery disease (CAD), in-hospital treatment, risk and severity of the ischemic event.

**Table 1:** – Baseline characteristics of the study population

Variables	Control (n = 26)	Training (n = 27)	p
**Demographics**			
Age, y	59±11	60±9	0.73
Gender M / F, n (%)	19 (73%) / 7 (27%)	20 (74%) / 7 (26%)	0.93
BMI, kg/m^2^	27.4±3.9	27.2±3.9	0.84
Hypertension, n (%)	17 (65%)	13 (48%)	0.21
Diabetes Mellitus, n (%)	10 (38%)	6 (22%)	0.84
Smoker, n (%)	5 (19%)	10 (37%)	0.15
Sedentarism, n (%)	26 (100%)	27 (100%)	1.00
Dyslipidemia	8 (31%)	9 (33%)	0.84
Previous AMI, n (%)	4 (15%)	3 (11%)	0.70
**Treatment**			
Aspirin, n (%)	26 (100%)	27 (100%)	1.00
Clopidogrel, n (%)	24 (92%)	27 (100%)	0.24
Ticagrelor, n (%)	2 (8%)	0 (0%)	0.24
β-blocker, n (%)	17 (65%)	23 (85%)	0.09
ACEI/ARB	16 (61%)	21 (78%)	0.20
**Event**			
STEMI, n (%)	14 (54%)	16 (59%)	0.51
NSTEMI, n (%)	12 (46%)	11 (41%)	0.69
Troponin, ng/mL (median, IQR)	22 (3-50)	32 (11-50)	0.35
**Risk Scores**			
TIMI STEMI score	3.3±1.8	2.7±1.8	0.38
TIMI NSTEMI score	3.7±1.3	3.2±1.1	0.31
GRACE (median, IQR)	129 (120-159)	139 (121-151)	0.74
**Treatment**			
Fibrinolysis, n (%)	4 (15%)	6 (22%)	0.73
Primary PTCA, n (%)	8 (31%)	8 (30%)	0.93
Pharmaco-invasive strategy, n (%)	3 (12%)	2 (7%)	0.66
BMS, n (%)	19 (73%)	23 (85%)	0.23
DES, n (%)	5 (19%)	1 (4%)	0.10

*Data expressed as mean ± SD, median (IQR, interquartile range; 95% CI) or number and percentage (%). Categorical data presented as numbers and percentages. BMI: body mass index; ACEI: angiotensin I converting enzyme inhibitor; ARB: angiotensin II receptor blocker; STEMI: ST-segment elevation myocardial infarction; TIMI: Thrombolysis in Myocardial Infarction Group; NSTEMI: Non-ST segment elevation myocardial infarction; GRACE: Global Registry of Acute Coronary Events Score; PTCA: percutaneous transluminal coronary angioplasty; BMS: bare-metal stent; DES: drug-eluting stent. P = NS for all comparisons (Student’s t, Chi-square or Fisher’s exact tests).*

The average adherence of patients in the TRAINING group to the exercise program was 28.0±6.8 sessions (minimum of 15 and maximum of 39 sessions), reaching an overall adherence of 88%.

### Cardiopulmonary exercise testing

Table 2 provides the CPX main data. After the exercise program period, the TRAINING group showed a significant increase in exercise duration, maximum workload achieved and peak VO_2._However, at the end of the four-month follow-up, no differences were observed in the variations of the parameters (delta [Δ]) measured between the groups (supplementary data III).

**Table 2:** – Standard echocardiographic and cardiopulmonary stress testing

Variables	Control			Training		
	
	Basal	4M	p*	Basal	4M	p*
**Echocardiogram**						
LA, mm	37.3±3.7	36.6±3.4	0.02	38.7±3.4	38.8±3.3	0.63
LVEDD, mm	50.2±4.3	50.0±4.3	0.21	50.9±3.1	51.3±3.5	0.38
LVESD, mm	33.1±5.0	31.9±3.9	0.05	33.6±3.0	34.6±4.6	0.11
LVEDV, ml	99.9±23.2	99.5±23.5	0.66	94.3±15.2	92.2±16.4	0.40
LVESV, ml	39.6±11.1	42.0±10.2	0.11	42.3±11.5	42.6±9.1	0.85
LVMI. g/m^2^	90.7±18.6	89.7±18.0	0.28	92.7±16.7	92.2±16.4	0.88
LVEF, %	61.3±5.7	60.0±5.1	0.19	60.0±5.9	59.6±5.6	0.70
WMSI	1.19±0.19	1.16±0.19	0.19	1.18±0.16	1.17±0.18	0.39
E Vel, cm/s	81±24	81±22	0.89	74±0.18	74±18	0.95
e’ Vel cm/s	7.1±1.6	7.2±1.8	0.80	6.9±1.7	6.9±1.8	0.89
E/e’ ratio	12.1±5.3	11.8±4.6	0.65	11.5±3.9	11.6±4.9	0.92
E/A ratio	1.21±0.52	1.13±0.39	0.34	1.09±0.41	1.20±0.46	0.16
**CPX**						
Exercise duration, secs	491±93‡	593±93	0.006	524±95‡	636±131	0.001
Basal HR, bpm	73±14	70±11	0.58	70±10	64±10	0.002
Max HR, bpm	133±22	134±15	0.84	127±19	128±19	0.78
Basal SBP, mmHg	124±11	119±14	0.35	120±18	117±14	0.51
Peak SBP, mmHg	169±18†	176±16	0.41	184±27†	178±18	0.41
Maximal exercise workload, W	109±43	119±48	0.11	126±49	151±65	0.007
Peak VO_2_, ml/kg/min	20.6±4.6	21.7±4.9	0.12	21.7±5.2	23.4±5.9	0.04
AT, ml/kg/min	12.3±2.3	13.7±2.4	0.08	12.3±3.2	14.3±3.4	0.11
VE, L/min	59.5±21.3	59.4±19.5	0.97	62.7±18.7	69.2±20.4	0.93

Data expressed as mean ± SD. LA: left atrium; LVEDD: left ventricular end diastolic diameter; LVFSF: left ventricular end systolic diameter; LVMI: left ventricular mass index; LVEDV: left ventricular end-diastolic volume; LVESV:left ventricular end-systolic volume; LVEF: left ventricular ejection fraction; WMSI: wall motion score index; E Vel: transmitral E wave velocity; e’ Vel:septal e’ wave velocity; A Vel: transmitral A wave velocity. HR indicates heart rate; SBP: systolic blood pressure; VO2: oxygen consumption; AT: anaerobic threshold; VE: minute volume. *Paired Student´s t test. ‡p=0.02, unpaired Student’s t test, for comparison with CONTROL basal. †p=0.04, unpaired Student’s t test, for comparison with.

### Conventional echocardiogram

Echocardiographic data are shown in Table 2 . A slight difference in the left atrial dimensions in the CONTROL group was noted at four months. No other significant differences were found between groups, including comparison of variations in all of these parameters.

### Analysis of LV contraction mechanics

#### Apical view

#### Strain and strain rate (SR)

No significant differences in strain or SR in response to exercise was found between the groups at the end of the follow-up ( Table 3 and Supplementary data IV).

**Table 3:** – Result of LV strain and strain rate analysis. Analysis of the apical window (longitudinal views, 4 and 2 chambers)

Variables	Control			Training		
	
	Basal	4 months	p*	Basal	4 months	p*
**APLAX**						
LS, %	17.5 ± 3.3	18.6 ± 2.5	0.09	17.6 ± 2.0	17.8 ± 2.8	0.54
LSR, 1/s	0.98 ± 0.16	1.04 ± 0.20	0.08	0.97 ± 0.15	0.95 ± 0.20	0.65
**A4C**						
LS, %	18.5 ± 3.3	18.6 ± 2.8	0.91	17.9 ± 2.7	17.8 ± 3.3	0.89
LSR, 1/s	1.01 ± 0.23	1.00 ± 0.20	0.98	0.93 ± 0.16	0.90 ± 0.21	0.44
**A2C**						
LS, %	18.7 ± 2.9	18.4 ± 2.7	0.36	19.4 ± 2.8	18.4 ± 3.4	0.06
LSR, 1/s	1.04 ± 0.21	1.01 ± 0.15	0.24	1.03 ± 0.17	0.92 ± 0.18	0.01
**GLOBAL**						
LS, %	18.3 ± 2.7	18.5 ± 2.5	0.55	18.3 ± 2.2	18.0 ± 2.9	0.48
LSR, 1/s	1.00 ± 0.18	1.02 ± 0.17	0.67	0.98 ± 0.14	0.92 ± 0.18	0.14

Data expressed as mean±SD. APLAX: apical window, longitudinal view; A4C: apical window 4-chamber view; A2C: apical window 2-chamber view; LS: longitudinal strain; LSR: longitudinal strain rate. P = NS for all comparisons (Student’s t test). *Paired Student´s t test.

#### Transversal axis

#### Circumferential and Radial Strain and SR, and Rotations

Data obtained from the analysis of LV transversal axis are presented in Table 4 . No significant differences in the variation of circumferential or radial strain and SR values were found between groups after the follow-up. Regarding rotational mechanics, at the end of the training period, the TRAINING group had lower values of basal rotation compared to the CONTROL group (-5.9±2.3 vs. -7.5±2.9^o^; p=0.03), with the delta of this parameter showing a borderline significance (p=0.05) between groups. Additionally, lower basal rotational velocity values were also observed in the TRAINING group (-53.6±18.4 vs -68.8±22.1 º/s; p=0.01), as presented in central illustration and supplementary data V.

**Table 4:** – Analysis of ventricular contraction mechanics obtained in the transverse left ventricular short axis (basal, mid and apical), left ventricular twist and torsion mechanics

	Control			Training		
	
	Basal	4 months	p*	Basal	4 months	p*
**PSAX – Basal**						
CS, %	17.0 ± 3.9	18.0 ± 3.1	0.11	16.6 ± 2.6	16.1 ± 3.4	0.38
RS, %	39.7 ± 21.0	34.3 ± 17.9	0.13	34.8 ± 13.9	31.8 ± 15.7	0.41
CSR, 1/s	1.54 ± 0.40	1.62 ± 0.36	0.42	1.52 ± 0.36	1.47 ± 0.31	0.58
RSR, 1/s	2.32 ± 0.92	2.41 ± 0.83€	0.68	2.20 ± 0.71	1.87 ± 0.71€	0.03
Rot,^o^	-6.7 ± 3.2	-7.5 ± 2.9†	0.18	-6.7 ± 2.9	-5.9 ± 2.3†	0.15
Rot vel,^o^/s	-66.6 ± 17.9	-68.8 ± 22.1‡	0.10	-57.8 ± 13.8	-53.6 ± 18.4‡	0.32
**PSAX – Mid**						
CS, %	15.8 ± 4.1	16.3 ± 3.6	0.55	15.9 ± 3.2	16.1 ± 3.8	0.71
RS, %	34.4 ± 19.3	39.4 ± 18.0	0.24	34.4 ± 11.9	35.6 ± 18.3	0.75
CSR, 1/s	1.44 ± 0.25	1.43 ± 0.26	0.75	1.43 ± 0.25	1.41 ± 0.31	0.48
RSR, 1/s	2.10 ± 0.84	2.31 ± 1.11	0.23	1.95 ± 0.58	2.16 ± 0.89	0.87
**PSAX – Apex**						
CS, %	19.3 ± 4.8	18.0 ± 3.9	0.16	20.5 ± 5.9	19.7 ± 5.8	0.62
RS, %	23.9 ± 9.0	19.1 ± 8.2	0.19	22.5 ± 7.7	25.0 ± 11.9	0.43
CSR, 1/s	1.53 ± 0.40	1.48 ± 0.42	0.67	1.56 ± 0.38	1.36 ± 0.39	0.09
RSR, 1/s	1.89 ± 0.97	1.80 ± 0.91	0.77	1.73 ± 1.18	1.90 ± 1.32	0.52
Rot,^o^	15.5 ± 6.5	15.5 ± 5.0	0.95	14.9 ± 5.6	14.6 ± 4.1	0.74
Rot vel,^o^/s	84.9 ± 26.8	81.1 ± 23.2	0.43	78.0 ± 21.7	73.8 ± 18.7	0.38
**GLOBAL**						
CS, %	17.4 ± 3.5	17.4 ± 2.6	0.95	17.7 ± 3.1	17.3 ± 3.6	0.62
RS, %	30.8 ± 12.7	30.7 ± 8.5	0.94	29.5 ± 8.8	30.1 ± 12.7	0.78
CSR, 1/s	1.51 ± 0.27	1.51 ± 0.21	0.97	1.51 ± 0.26	1.41 ± 0.27	0.21
RSR, 1/s	2.10 ± 0.58	2.17 ± 0.52	0.57	1.96 ± 0.47	1.98 ± 0.74	0.92
**TORSION**						
*Twist ,* ^*o*^	22.2 ± 7.1	23.1 ± 6.0	0.46	21.6 ± 6.2	20.5 ± 3.9	0.41
Tw vel,^o^/s	145.5 ± 32.3	149.9 ± 35.9¥	0.53	135.8 ± 28.3	127.4 ± 32.2¥	0.28
Torsion,^o^/cm	2.6 ± 0.8	2.8 ± 0.8£	0.18	2.6 ± 0.7	2.4 ± 0.4£	0.27
T vel,^o^/s.cm	17.4 ± 3.9	18.5 ± 5.2	0.22	16.3 ± 3.6	15.0 ± 3.6	0.17

Data expressed as mean±SD. PSAX-basal: parasternal short axis, basal; PSAX-mid: short axis, mid level; PSAX-apical: short axis, apical level; CS: circumferential strain; RS: radial strain; CSR: circumferential strain rate; RSR: radial strain rate; Rot: rotation; Rot Vel: rotational velocity; Tw vel: twist velocity; T vel: torsional vel. *Paired Student´s t test. €p=0.01, unpaired Student’s t test, for comparison with CONTROL 4M. †p=0.03, unpaired Student’s t test, for comparison with CONTROL 4M. ‡p=0.01, unpaired Student’s t test, for comparison with CONTROL 4M. ¥p=0.02, unpaired Student’s t test, for comparison with CONTROL 4M. £p=0.02, unpaired Student’s t test, for.

**Central Illustration:** : Influence of Physical Training after a Myocardial Infarction on Left Ventricular Contraction Mechanics

LV basal rotation and rotational velocity. LV, left ventricle (central illustration). * Paired Student´s t test. ** Unpaired Student’s t test, for comparison of deltas of values between groups after follow-up. *** Unpaired Student’s t test. (Original figure, created by the authors).

## LV twist and torsion

Results of the LV twist and torsion analyses are shown in Table 4 . At the end of the 4-month follow-up, the TRAINING group showed significantly lower values of twist velocity (127.4±32.2 vs. 149.9±35.9 º/s; p=0.02) and torsion (2.4±0.4 vs. 2.8±0.8º/cm; p=0.02). However, none of the deltas of all torsional mechanics parameters achieved a statistically significant difference (supplementary data VI).

## Correlation analysis

Pearson’s linear correlation were performed to assess correlation between the VO_2_deltas (four months vs. baseline) obtained from CPX, and the deltas of several echocardiographic parameters. There were only poor and non-significant correlations (supplementary data VII).

## Discussion

This present study investigated the hypothesis that cardiovascular rehabilitation by a supervised exercise program would have a positive impact on the LV contraction mechanics in a population after an uncomplicated AMI. There is a lack of knowledge, with few studies investigating the real benefit of exercise on LV remodeling, with analysis of LV contraction mechanics in this population. To date, to the best of our knowledge, this is the first study to investigate this hypothesis in such a detailed way, considering the large number of LV contraction parameters including those of LV systolic mechanics analyzed in our study. In this work, we used the speckle tracking to further investigate LV systolic function. This technique has being proved superior to LVEF in myocardial contraction assessment, as it is less susceptible to hemodynamic conditions, more reproducible,^38^ and also a better prognostic factor for cardiovascular events in a wide spectrum of cardiac diseases.^39 - 41^ This is particularly important after a myocardial ischemic event where an accurate LV systolic analysis is essential.

Regarding longitudinal, circumferential and radial myocardial deformations, in general, the TRAINING group did not have a superior contraction performance over the CONTROL group up to 4 months post-AMI. Although the TRAINING group showed a significant increase in exercise duration, maximum workload achieved and peak VO_2_after the training period, no increment in longitudinal or transversal LV mechanics was observed. However, we identified a very interesting finding in relation to the LV torsional mechanics. Compared to the CONTROL group, the TRAINING group showed significantly lower values of rotation and rotational velocity of the LV basal segments, as well as lower values of twist velocity, torsion and torsional velocity after the 16-week training period. McGregor et al.^42^ observed similar results in their elegant exploratory study.^42^ These authors also described a reduction in LV twist and twist velocity after 10-weeks of physical training sessions, twice a week, in a similar population who had an AMI and still maintained a preserved LV function (LVEF > 50%). In their study, this final result on LV twist was linked to a reduction in both basal and apical rotations. Finally, as similar to our study, they did not find a significant positive impact of exercise on LV strain (longitudinal, circumferential or radial).

Extrapolating to highly-trained athletes, despite some contrasts in the findings, studies point to a common and final real impact of exercise on LV torsional mechanics. Stöhr et al. described a reduction of apical rotation and LV twist in individuals with high aerobic fitness.^43^ The same was found by Nottin et al.^44^ studying elite cyclists, and Zócalo et al.^45^ assessing professional soccer players. A reduction of LV basal and apical rotations rates, and torsional rate, were described. Weiner et al.^46^ also showed interesting findings with competitive rowing athletes. They described a so called “phasic phenomenon” in a program of high level physical activity, comprised by an acute phase of augmentation of LV twist, followed by a subsequent and chronic reduction of this parameter.^46^ Based on this, one may postulate that a greater enhancement of LV twist during exercise may represent greater systolic efficiency systole in these individuals. This outcome may be interpreted as a “LV torsional reserve” in athletes and in individuals who exercised post-AMI, representing a physiologically more efficient torsional mechanics, which could lead to an increment in functional capacity, and perhaps to a better clinical course.

The most accepted design of the myocardial muscle architecture is that proposed by Torrent-Guasp et al.,^47 , 48^ who described the heart as a muscle band “folded” in double helix. In terms of energy expenditure, it provides a more efficient form of contraction, and a more homogeneous distribution of cavity wall stress, with less myocardial oxygen consumption, compared to a simple radial LV cavity deformation.^49 , 50^

The effect of exercise on LV strain is still unclear. In a systematic review and meta-analysis, Murray et al.^51^studied the effect exercise on LV global longitudinal strain across a wide range of healthy, at risk of cardiovascular disease, and chronic diseased populations. A moderate effect of exercise was observed in individuals with heart disease compared to non-exercising controls, and no significant effect of exercise was observed in individuals at risk or healthy populations. As in our study, McGregor et al.^42^ did not find a positive impact of exercise on longitudinal strain. So, despite the good accuracy and more robust data in the literature on longitudinal strain, LV twist could be a more sensitive parameter for evaluating LV global systolic response to physical exercise in this population.

### Limitations

First, the relatively small sample size, the short duration of the exercise program, the small number of training sessions for patients in the TRAINING group (twice a week), and the low adherence of some patients to the program may have decreased the power of the study to demonstrate possible intergroup differences. Subjects were encouraged to attend the sessions, and those who attended less than one session per week, or stopped the sessions, were excluded. A sample size of 72 participants, as calculated, was not achieved, and this is also a limitation. In addition, besides exercise, no other health instructions, such as nutritional or psychological support were given, which could have affected the final result. No data concerning chronic coronary syndromes or peripheral artery disease were collected, which may have had an impact on exercise. Finally, not all data derived from the CPX, including data on the respiratory exchange ratio, were addressed in this study - these could have revealed some differences between groups.

Another important point is the subjectivity of the echocardiogram which can lead to quantification bias. Small variations in the acquisition at LV apical level, which do not have an anatomical marker, can result in distorted values. Images were acquired from the apical short-axis view when the visibility of the myocardial segments was clear. Additionally, we created another criterion to ratify the correct acquisition, which was the visualization of at least a trend of a counterclockwise rotation of these segments, which would be physiologically expected. Finally, it is important to remind that the examiner was blinded to the patient’s group allocation.

## Conclusion

In our study, exercise led to no significant improvement in the LV longitudinal, radial and circumferential myocardial deformation parameters. However, exercise was associated to a reduced basal rotation, twist velocity, torsion and torsional velocity. This outcome may be interpreted as a “LV torsional reserve” in individuals who exercised in post-AMI, suggesting a physiologically more efficient torsional mechanics.

## * Supplemental Materials

For additional information Supplemental Material 1, please click here .

For additional information Supplemental Material 2, please click here .

For additional information Supplemental Material 3, please click here .

For additional information Supplemental Material 4, please click here .

For additional information Supplemental Material 5, please click here .

For additional information Supplemental Material 6, please click here.

For additional information Supplemental Material 7, please click here .
